# 3D-printed models in the courtroom: mock-jurors’ perceptions and experiences of 3D-printed models of human skeletal remains

**DOI:** 10.1080/13218719.2025.2556459

**Published:** 2025-10-08

**Authors:** Hannah Fawcett, Jessica Ball, Jessica Glynn, Aminah Barnes, Matthew Brooks, Rachael Carew, David Errickson

**Affiliations:** ^a^ School of Psychology, Manchester Metropolitan University, Manchester, UK; ^b^ School of Life Sciences, Coventry University, Coventry, UK; ^c^ Cranfield Forensic Institute, Cranfield University, Cranfield, UK

**Keywords:** 3D-printed models, forensic evidence, juror wellbeing, jury decision making, physical trauma evidence, trauma-informed court practices, trauma-informed legal systems

## Abstract

The use of 3D-printed models of skeletal remains has notably enhanced jurors’ comprehension of forensic evidence and skeletal trauma, enabling clearer interpretations and diminishing the confusion associated with expert terminology. However, the hands-on tactile sensory engagement with these highly realistic 3D-printed models of human remains could be more distressing than traditional passive modalities. In the first study, to qualitatively examine jurors’ perceptions of 3D-printed skeletal remains as evidence, mock-juror participants read a written summary of a murder trial before interacting with an accurate 3D-printed model of a real murder victim’s skull showing evidence of gunshot trauma. The deliberations were audio-recorded and subjected to reflective inductive semantic thematic analysis, which identified four critical themes: ‘Realistic and creepy’, ‘Overcoming uncomfortableness’, ‘Illustrative tool’ and ‘A piece of the puzzle’. The findings revealed that 3D-printed models have an emotional impact on jurors and indicate that caution should be exercised in their use in courts.

## Introduction

In the courtroom, forensic experts often need to elucidate complex medical evidence that demonstrates injuries such as blunt force trauma, sharp force trauma and ballistic (gunshot) trauma to a victim’s bones (Carew et al., 2021). Hearing oral evidence is a passive process with jurors often confused by complex medical and scientific terminology (Eldridge, 2019), meaning visual aids can be crucial in facilitating jurors’ comprehension of this unfamiliar testimony and evidence (Blau et al., 2019; Errickson et al., 2014) and enhancing the perceived credibility of the presenting expert witness (Wilcox & NicDaeid, 2018). Photographs have traditionally been used to demonstrate victim injuries and enhance jurors’ understanding, whilst mitigating the practical and ethical issues associated with introducing actual human remains into the courtroom (Carew et al., 2019; Errickson et al., 2020; Rempel et al., 2019). However, this approach has received criticism in that errors and incorrect interpretations can occur in the process of translating two-dimensional photographs into three-dimensional mental maps (Sheppard et al., 2020). Furthermore, the often-graphic nature of autopsy photographs could be upsetting for jurors, enhance sympathy for the victim and ultimately affect the accuracy of their decision making (Bandes & Feigenson, 2021; Haragi et al., 2020). It is argued (Henningsen et al., 2023) that three-dimensional aids in the form of 3D-printed models of skeletal remains, and the mapping of injury images onto 3D mannikins, help to limit the distress caused by autopsy imagery. 3D-Printed models and 3D mannikins are becoming increasingly commonplace in legal practice around the world with their use documented in England, Germany and the United States (see, for example, Saunders, 2018; Scott, 2016). Increased media attention is being directed to 3D-printed models in particular (University of Portsmouth, 2023) despite limited evidence of their impact upon jurors’ wellbeing.

### The use of 3D-printed models of skeletal remains

3D-printed models are a valuable extension of 3D-visual imaging in forensic settings (Carew & Errickson, 2019; Jani et al., 2020). Indeed, 3D-printed models of human skulls have been provided as illustrative aids in courts around the world since 2009 (Kettner et al., 2011). Forensic experts need to demonstrate fine features to explain the force required to produce the injury sustained (Carew et al., 2021), detail fracture lines (Barrera et al., 2019), illustrate characteristics of gunshot entry and exit wounds (Carew et al., 2021) and display distinctive tool mark patterns (Rutty et al., 2013). 3D-printed models can be non-invasively produced, meaning the integrity of the original skeletal material is maintained, and burial can occur (Errickson et al., 2017). 3D-printed models can also be duplicated at different magnitudes to enlarge and enhance visibility of key trauma features (Edwards & Rogers, 2018). Nevertheless, caution must be applied regarding the limits of this technical procedure as it has been noted (Carew et al., 2021) that not every nuance, particularly fine fracture lines, is guaranteed to be successfully captured within 3D-printed models. Despite this limitation in utilising 3D-printed models as demonstrative evidence, they have been used as illustrative aids in courtroom proceedings in many countries including England and Wales (Baier et al., 2018; Scott, 2016), Canada (Halterman, 2015), Germany (Kettner et al., 2011), Poland (Woźniak et al., 2012) and the United States (Baier et al., 2018).

The integration of technology into judicial processes represents a shift from oral to visual modes in the presentation of evidence and caters to increasingly visually literate and media savvy jury pools (Schofield, 2009; Schofield & Fowle, 2013). Despite their use in courtrooms, there are currently no clear standards for creation of 3D-printed models for this purpose, or guidance as to what should and should not be viewed in open court. Thus, the prevailing research has focused on ascertaining the best technical specifications to produce 3D-printed models to support model accuracy and juror comprehension. Carew et al. (2021) advocate that only accurate 3D-printed models that effectively represent the relevant forensic details should be employed as evidence in courtrooms, and subsequently developed a flexible protocol developed to evaluate the accuracy of 3D-printed models (Carew et al., 2022) as a way of ensuring their reliability as courtroom evidence (although it has not been formally adopted). However, this remains a discretionary protocol, rather than a legally binding rule.

In addition to the technical limitations of 3D-printed models, concerns have been raised over the ethics of using 3D-printed models in the courtroom, as well the ethics of ownership, storage and disposal of the models (Errickson et al., 2017). More specifically, it has been argued (Hirst et al., 2020) that thorough examination of the potential emotional ramifications for jurors needs to occur, given the high realism of 3D-printed models. Unlike photographs and three-dimensional imaging, 3D-printed models allow jurors to touch, feel, rotate, orientate into anatomical position and physically mimic the causation of injury (Barrera et al., 2019; Bizzotto et al., 2016; Carew et al., 2019). Therefore, 3D-printed models add levels of spatial and haptic information that are lacking in other evidence modalities such as photographs, and facilitate a more active, direct and personal engagement with a victim’s injuries (Carew & Errickson, 2020).

### Effect on decision making of 3D-printed models of skeletal remains

The use of computer animations/interactive virtual simulations, in which virtual environments can be created from crime scene survey data and victim remains, is superior in conveying complex evidence, allowing jurors to improve concentration, enhance comprehension and better assimilate information (Schofield, 2009). Such use of visual evidence presentation systems, including digital displays, computer-generated graphical presentations and three-dimensional simulations, has been introduced as jurors often struggle with the complexity of technical evidence presented in trials, particularly as it falls outside of their everyday experiences (Schofield & Fowle, 2013); orally presenting significant volumes of dense and technical evidence can overwhelm and confuse jurors, producing feelings of boredom and frustration (Schofield, 2009). Certainly, 3D-printed models may provide clarity to expert testimonies and reduce confusion of complex terminology compared to photographs and verbal testimony alone (Blau et al., 2019).

Compared to receiving solely verbal testimony, examining 3D-printed models significantly improves mock-jurors’ comprehension of skeletal trauma evidence (Errickson et al., 2020). Moreover, in comparison to alternative computer visualisation techniques, 3D-printed models improved jurors’ understanding of technical language, which in turn better informed their decision making (Errickson et al., 2020). The finding that 3D-printed models enhance juror comprehension is supported by psychological theories, such as the multimodal learning theory (Bouchey et al., 2021; Massaro, 2012), which posits that people learn more effectively when information is presented using multiple channels. In the context of juror comprehension, 3D-printed models add a tactile dimension to the audio description of the case and allow for visual observations. Moreover, a realistic 3D format, compared to a flat photographic presentation, may enhance jurors’ ability to accurately visualise injuries and skeletal features and so further facilitate accurate evidence comprehension and decision making.

According to the cognitive load theory (Sweller, 1988, 2011) by providing a tangible reference point, 3D-printed models should reduce the cognitive load required for imagining spatial and physical properties of the evidence and should therefore improve comprehension of verbal forensic evidence (Blau et al., 2019; Schofield & Fowle, 2013). Moreover, the hands-on engagement with 3D-printed models, compared to oral evidence, requires active rather than passive engagement from jurors, which is associated with increased attention and memory (Cooper, 1999; Harston, 2008). This means 3D-printed models may be powerful instruments that better equip jurors towards reaching a well-informed verdict (Baier et al., 2017).

In contrast to the above benefits of 3D-printed models, there is a risk that jurors may place too much emphasis on 3D-printed models to the detriment of other evidence, and fair decision making. The ‘CSI effect’ refers to how the viewing of crime and forensic-based television shows can lead to unrealistic expectations of forensic evidence in real-life court cases (Alldredge, 2015; Call et al., 2013). In line with the ‘CSI effect’ (Christoloukas & Mitsea, 2022), introducing ‘scientific’ 3D-printed models in court could be problematic as jurors hold forensic science evidence in such high regard that the presence of a forensic evidence rather than the overall quality of the case against the defendant could lead to a guilty verdict (Alldredge, 2015). Thus, the mere presence of a 3D-printed model could feed into jurors’ over-reliance on scientific evidence and bias jurors to contrasting evidence. However, the ‘CSI effect’ is disputed, with arguments (Klentz et al., 2020) suggesting there is little evidence to show it exists. It may be that jurors find the quantity of evidence presented to be more influential in reaching a verdict than the type of evidence provided (Hawkins & Scherr, 2017). This implies that the guilty verdicts may be more common in the presence of 3D-printed models as they add to the body of prosecution evidence available to jurors, rather than that jurors are blinded by science. Nonetheless, the evidence once again focuses on the decision making of jurors, neglecting to consider the emotional impact for jurors viewing such evidence. The strengths and limitations of presenting in court 3D-printed models of human skeletal remains are summarised in Table 1.

**Table 1.** Strengths and limitations of 3D-printed models of human skeletal remains as demonstrative evidence in court.

Strengths	Limitations
Increased attention to, and understanding of, accompanying oral evidence	Potential emotional distress
Illustration of fine features	Some nuance of detail can be lost
Preservation of the integrity and dignity of original remains	No agreed technical specifications for admissibility
Permits analysis post burial	Potential disproportionate influence on jurors’ decisions (‘CSI’ effect)
Magnification of fine detail	Potential for disproportionate increase in victim empathy
Multi-sensory engagement	No clear protocols for 3D-printed model storage, use and disposal
Enhanced understanding of spatial and physical elements	
Potential increase in guilty verdicts	

### The emotional impact of 3D-printed models of skeletal remains

The potentially distressing nature of 3D-printed models of human remains is not universally acknowledged, with some suggesting that they are a ‘minimally graphic’ form of evidence (Edwards & Rogers, 2018). Thus, the ethical sensitivity shown towards post-mortem photographs may not be consistently applied by practitioners to 3D-printed models (Carew & Errickson, 2020), despite arguments that 3D-printed models should be treated with the same ethical considerations as human remains (Campanacho & Alves Cardoso, 2019). Understanding the emotional impact of 3D-printed models would facilitate best practice for the use of these models in courtrooms, as well as enable the development of ethical standards for the treatment of 3D-printed models of human remains in general.

The presentation of gruesome images in courtrooms can lead to jurors experiencing emotional distress, which can predispose them to attribute guilt to the defendant (Bright & Goodman-Delahunty, 2006, 2011). This is supported by the capable control model (Alicke, 2000), which suggests that the negative emotional arousal elicited by gruesome photographs manifests in a desire to find someone culpable for the injury, and thus a higher likelihood of guilty verdicts, regardless of the strength and quality of other case evidence. This is particularly important when considering the nature of the photographs; coloured photographs in comparison to black and white photographs lead to greater emotional distress in jurors’ (Salerno, 2017), implying that colour adds realism to the images. Despite recent research into 3D-printed human remains as courtroom evidence (e.g. Carew et al., 2023) it is unclear whether the life-like nature of 3D-printed models corroborate the capable control model (Alicke, 2000), or whether they are less graphic than images of actual remains, which enable more informed and reasoned decision making (Edwards & Rogers, 2018; Errickson et al., 2020).

Although it has been proposed that 3D-printed models are not graphic (Edwards & Rogers, 2018), the technical advancements in three-dimensional printing processes have allowed 3D-printed models to be regarded as highly realistic (Carew & Errickson, 2019), even if they can lack some fine details (Carew et al., 2021). The possibility of producing more realistic 3D-printed models through the addition of skin colour has been proposed (Villa, 2017). However, others (Errickson et al., 2014; Haragi et al., 2020; Sheppard et al., 2020) caution against this approach, suggesting that 3D-printed models could become too realistic or disturbing for jurors, who prefer less realistic evidence. More recent research (Henningsen et al., 2023) shows conflicting views, with some Danish judges, lawyers and forensic pathologists seeing 3D-printed skulls as equivalent to skeletons shown in museum exhibits, and others believing 3D-printed human remains to be emotionally confronting. What was agreed upon was that jurors may find 3D-printed models more emotionally charged than equivalent photographs.

A previously unexplored concept is that the realistic nature of 3D-printed models of victims’ remains may facilitate greater empathy and identification with the victim. There are both cognitive and emotive aspects to empathy (Bandes & Feigenson, 2021), with Davis (1983) defining empathy as the cognitive and emotional understanding of another’s experience developed from considering their perspective. Research demonstrates that empathy towards victims (Bottoms et al., 2014; Lilley et al., 2023) and defendants (Curley et al., 2022) can unduly impact juror decision making. Indeed, Wood et al. (2014) found that high empathy towards the defendant was associated with jurors viewing the defendant as being more remorseful and less responsible for the offence and was related to a tendency towards not guilty verdicts, compared to low situational empathy. The role of empathy in juror decision making is also recognised by lawyers, who use examination and cross examination techniques to actively manipulate juror empathy towards witnesses (Stevenson et al., 2009). This evidence illustrates the need for careful consideration before introducing 3D-printed models of human remains into court given that they could actively manipulate juror empathy, emotions and decision making. A case-by-case evidence-based evaluation of the benefits and limitations of utilising this type of evidence is needed to illustrate whether the enhanced juror comprehension associated with 3D-printed models of human remains outweighs negative legal and ethical considerations (Sheppard et al., 2020).

### Juror distress

It is well established that symptoms of secondary trauma amongst jurors can follow exposure to graphic evidence (L. Miller, 2008; M. K. Miller & Bornstein, 2004) and harrowing details of traumatic events experienced by victims (Hensel et al., 2015). This indirect trauma can trigger emotional distress and symptoms of post-traumatic stress disorder symptoms (PTSD; American Psychiatric Association, APA, 2013), including anxiety, depression, flashbacks and nightmares, with an international systematic review illustrating that these symptoms occur in up to 50% of jurors (Lonergan et al., 2016). Jurors in both England and Wales (Robertson et al., 2009) and Scotland (Welsh et al., 2020) have also been shown to experience distress following their jury service with a range of stressors cited including disturbing evidence, deliberations and deciding a verdict. Failure to provide jurors with wellbeing support may result in jurors experiencing these symptoms for decades (Barak & Szor, 2000), and specialised dedicated support for jurors’ wellbeing is lacking in England and Wales (Brooks et al., 2025) and many other jurisdictions around the world (such as Canada and many states in the United States). If 3D-printed models are indeed as distressing as other forms of graphic imagery, they may cause a significant emotional burden and a detrimental effect on jurors’ mental wellbeing and impact their decision making. The potential ramification of exposure to 3D-printed models of human remains are concerning, hence it is paramount that jurors’ mental health be at the forefront of decisions to utilise such evidence in courtrooms.

### The current study

It is of critical importance that the impact of 3D-printed models upon jurors is understood. The dearth of evidence surrounding the utilisation of 3D-printed models in courtrooms in general, and specifically upon the emotional wellbeing of jurors, could be deemed negligent (Kettner et al., 2011; Scott, 2016; Woźniak et al., 2012). Understanding the impact of this evidence will facilitate achieving a balance between juror comprehension and juror wellbeing and help understand the support needs of jurors exposed to 3D-printed evidence in court. Consequently, the present research qualitatively investigates mock-jurors’ attitudes, emotions and experiences of engaging with 3D-printed models of skeletal remains in the context of a mock-homicide case to address the following research questions:

1. What are (mock) jurors’ interpretations of 3D-printed models of skeletal remains within homicide court cases?
2. What are (mock) jurors’ emotional reactions to 3D-printed models of skeletal remains within homicide court cases?

The emotional impact of the 3D-printed model includes how it affects perceptions and experiences of stress, distress, anxiety, uncomfortableness and upset. Interpretations of the model relate to perceptions of the 3D-printed model’s accuracy, usefulness, reliability and role in decision making. Given the small-scale qualitative approach of the research, the verdicts reached by each jury are not of central interest, rather the way in which the 3D-printed model is used within the deliberations is of importance to the research.

## Method

### Participants

The sample consisted of 13 female undergraduate students aged between 18 and 48 years (*M* = 23.62, *SD* = 8.75) taking part in small mock-juror deliberations. The study did not seek to specifically recruit only female participants but used an opportunity sample of individuals recruited from the host institution via responses to campus posters and advertisements on the university’s student research portal. As the discussions and participants’ reactions and perceptions of the 3D-printed model, rather than the verdicts, were of interest to the research, the small juries facilitated rich in-depth discussions and a high degree of contact with the 3D-printed model. Student participant decision making has been shown to correspond with that of the wider public in mock-juror studies (Bornstein et al., 2017), and the qualitative nature of this study justifies the focus on richness of data over larger sample size (Hammersley, 2015; Park et al., 2017). All participants were eligible to participate in jury service in England and Wales, as defined by the Juries Act (1974), although none of the participants had completed jury duty previously.

### Materials

The 3D-printed model used in the study was created by Carew et al. (2021) and depicted an adult skull, which showed evidence of gunshot trauma in the form of bullet entry and exit wounds. The model was generated using post-mortem computed tomography (PMCT) data in accordance with appropriate guidance (Carew et al., 2019). The bullet entry and exit wounds were visible on the right parietal bone and the left temporal bone, respectively, and bone flaking and radiating fractures were also evident.

Consistent with much mock-juror research (Fawcett, 2016; Henningsen et al., 2023; Kettner et al., 2011; Matsuo & Itoh, 2016), participants were presented with a transcript of a murder trial. Attempts were made to simulate a real court case by using a real case as the foundation of the materials and presenting evidence in the form of a mock transcript (rather than summary) that followed the order of evidence presentation seen in real court practice (opening statements, prosecution case, defence case, closing arguments, homily). Brackets were used within the transcript to enhance the realism of the case through outlining the supposed movement of different individuals within the courtroom (e.g. *[The usher accompanies the witness out of the courtroom, before returning and taking their seat.]).*

The transcript was 20 pages in length and based on a real-life case, although details were changed to maintain anonymity of those involved. The transcript featured a female defendant accused of the murder of a male victim by means of a single gunshot wound to the head. The evidence presented by the prosecution included the victim’s mother who testified that the relationship between her son and his girlfriend was unstable, as well as the mother’s belief that her son would not have ended his own life. A forensic expert presented the 3D-printed model evidence. They explained that the 3D-printed model was created by CT scanning of the victim’s skull, which was a reliable technique, conducted by a trained professional. They further discussed that the direction of the bullet and the skeletal injury did not correlate with suicide. A blood splatter and fingerprint analysis expert concluded the lack of fingerprint and blood splatter at the crime scene suggested the crime scene had been tampered with. Finally, a medical expert concluded that the victim had died hours prior the police arriving. In contrast, the defence argued the victim died by suicide, with arguments focusing on evidence of mental health challenges experienced by the deceased prior to their death. Furthermore, the defence contested the time of death presented by the medical expert. Cross examination of the forensic expert involved the admission that 3D-printed models are not always 100% accurate in replicating all details.

### Procedure

The study adhered to the British Psychological Society’s (BPS) Code of Human Research Ethics (2021) and received Institutional ethical approval. A convenience sample of participants was recruited through posters and notifications on the university research participation website, and all participated between February and March 2022 on campus at Manchester Metropolitan University. Those interested in participating emailed Author 2 (female dissertation student) who allocated them to a deliberation group dependent on their availability for the scheduled sessions and who was solely responsible for data collection. All those that expressed interest participated, and there was no drop out. Deliberation groups consisted of two to four participants (see Table 2) who were unknown to the researcher and understood the project to be a psychology dissertation research project. On arrival participants read the participant information sheet and completed necessary informed consent and were given an opportunity to ask questions. Once consent was provided, participants were each given a printed copy of the criminal murder trial transcript to read at their leisure. Once all participants indicated that they had read and understood the trial transcript the audio recording of the sessions commenced. Participant groups were presented with one 3D-printed model of the victim’s skull to examine. Participants were asked to deliberate in their jury group to reach a unanimous decision regarding the defendant’s innocence or guilt. The 3D-printed model and trial transcript were retained by the group during the deliberations to facilitate discussion of this evidence, and all deliberations were audio recorded with a Dictaphone. The researcher left the room whilst deliberations took place, and participants were free to organise deliberation discussions as they pleased. Jury groups were concluded when the groups had reached their decision, at which point the researcher was invited back into the room to hear the group verdict, and the audio recordings stopped. All participants were thanked and debriefed at the conclusion of the study, and sources of support were provided.

**Table 2.** Jury deliberation groups and participants’ pseudonyms and ages.

Jury	Name	Age (years)
1	Katie	18
	Georgia	19
	Jane	21
2	Holly	22
	Charley	19
3	Hannah	20
	Jessica	21
	Alison	21
	Isobel	19
4	Tegan	21
	Karen	37
	Stacey	48
	Elsie	21

### Data analysis

The mock-jury deliberations ranged from 12 to 23 minutes (*M =* 17 minutes and 15 seconds, *SD =* 4 minutes and 30 seconds). Although short, the deliberations yielded detailed and novel data on the participants’ perceptions of 3D-printed models with the themes evident across the different deliberation groups. It was not the case that shorter sessions yielded fewer insights, rather that the range of evidence discussed was more focused in the shorter deliberation groups. The audio recordings were transcribed and analysed using a reflexive, inductive, semantic thematic analysis to examine the participant’s experiences and perspectives on the 3D-printed model (Braun & Clarke, 2022). Participants were assigned pseudonyms. Transcription formed part of the familiarisation process for the analysis by Author 2 who also instigated the coding of the data. All data analysis was conducted manually by hand with notes and comments made in the margins of transcripts. Coding was deemed complete when we deemed that the themes were detailed, rich, independent and informative enough that no further coding was necessary (Braun & Clarke, 2021).

Initial open coding resulted in candidate themes – for instance, negative emotions towards 3D-printed models. These candidate themes were reviewed and refined with Author 1 via discussion and review of transcripts; initial codes were grouped together thematically by physically grouping printed quotes and author comments. Theme names were constructed during analysis and based on the inherent thematic content of the themes, rather than based on any pre-existing categories. No conflict emerged in the naming of themes. The study took various steps to ensure rigour (Shenton, 2004), including using an established method of data collection (mock-juror deliberations; see for example, Charron & Woodhams, 2010; Fawcett & Clark, 2015; Richardson et al., 2025; Wiener et al., 2011) and employing a process of reflexive analysis throughout the process of data collection, transcription and analysis. Bracketing was implemented through data analysis being conducted by members of the research team who did not have experience of generating and utilising 3D-printed models in their professional practice, as well as reflexive journalling throughout these processes by those collecting and analysing data (Ahern, 1999).

As a research team we have a varied professional backgrounds including juror decision making, forensic anthropology and psychological trauma. Given the dearth of research on this point and a lack of direct juror experience amongst the research team, we approached this project with no clear perception of how 3D-printed models of human remains may be perceived, or if they may negatively affect wellbeing at all. Our desire to understand the experience of this type of evidence led us to adopt a mock-jury qualitative approach, and our choice of thematic analysis reflected our belief that we would be able to ‘identify, analyse and report patterns evident within the data’ (Braun & Clarke, 2006, p. 79).

## Results

### Analysis

Reflexive, inductive, semantic thematic analysis as outlined by Braun and Clarke (2022) was utilised whereby themes are conceptualised as stories within the data that are generated using a combination of the researcher’s skill and theoretical assumptions and the data itself, arrived at via careful analysis and continuous reflexivity. The analysis took the format of familiarisation with the data via transcription and (re)reading, highlighting key words and phrases, grouping related ideas and generating appropriate theme names. This process generated four key themes in the data: (a) Realistic and creepy, (b) Overcoming uncomfortableness, (c) An illustrative tool, and (d) A piece of the puzzle.

#### Theme 1: Realistic and creepy

In the initial stages of deliberations, the focus of all mock-jurors was primarily on the 3D-printed model of the victim’s skull, to which participants voiced varied cognitive and emotional reactions to the evidence. Holly (Jury 2, Line 17) referred to feeling ‘*scared’* to pick up 3D-printed model, with a fellow mock-juror describing the 3D-printed model as *‘creepy’* (Stacey, Jury 4, Line 2). Karen cited the 3D-printed model to be unsettling due to the knowledge that it represented a real victim’s skull: ‘*If we were not told that it was someone’s or like a real person, then I would not have considered it to be creepy, but the fact that I know that it is someone’s actual head, I think that it is’* (Karen, Jury 4, Line 3–4). Similarly, Jessica exclaimed, *‘I can’t believe that is someone’s actual skull’* (Jessica, Jury 3, Line 1). Thus, although clearly aware that the 3D-printed model was different to the victim’s actual skeletal remains, as it *‘would be disgusting if that was real’* (Karen, Jury 4, Line 13–14), the 3D-printed model was seen as not only *‘very realistic’* (Katie, Jury 1; Line 1) but as emotionally and ethically equivalent to actual human remains. In general, participants were impressed by the technology underpinning the model’s creation: *‘It is amazing, isn’t that 3D printing’* (Elsie, Jury 4, Line 12). These reactions imply that it is not merely the print itself, but the knowledge that it is an accurate representation of a victim’s skull that makes it *‘creepy’* and led to discomfort among the mock-jurors.

#### Theme 2: Overcoming uncomfortableness

There was a sense of shock amongst the participants at first viewing the 3D-printed model: *‘Oh my god, I thought that it was going to be a picture (laughing)’* (Holly, Jury 2, Line 1). The uncomfortableness the 3D-printed model caused was evident in the reluctance to physically handle the 3D-printed model shown by some participants: *‘That’s the hole, I don’t want to pick the skull up I am scared, oh yeah that’s the hole where it went in’* (Holly, Jury 2, Line 17–18). Likewise, Jan (Jury 1, Line 3) states: *‘I’m trying to be careful with it, I just don’t like the feel of it’*. All participants except one selected impersonal impartial terms such as ‘*physical evidence’*, *‘it’* or ‘*the skull’* when referring to the 3D-printed model. Karen was the only participant to refer to the 3D-printed model by the victim’s name: *‘So, this is Tommy. . . .* *There you go, go ahead, have Tommy for me. . . .* *Can you help turn Tommy for me’* (Karen, Jury 4, Line 32). Given that participants clearly viewed the skull as realistic and unsettling (see ‘realistic and creepy’ theme), this use of non-humanising language, rather than the victim’s name, may be an active attempt to suppress the uncomfortableness elicited by the model by removing some of its humanity.

In contrast to the negative responses to the 3D-printed model, one participant, though shocked at the model, highlighted that laughing was an inappropriate response: *‘Don’t laugh it’s not funny. I was not expecting it be someone’s actual head’* (Charley, Jury 2, Line 2). This suggests nervous laughter was not unanimously seen as a suitable coping mechanism by all the mock-jurors. This varied response to the 3D-printed model indicates that the mock-jurors experienced a spectrum of emotional and psychological responses. There were obvious signs of discomfort, and perhaps trauma-responses to the evidence, whilst some mock-jurors presented a more detached manner. This highlights the potentially diverse impact of such evidence in a courtroom and the potential role of experience and individual differences in shaping jurors’ reactions to this type of evidence.

#### Theme 3: Illustrative tool

During the mock-jury deliberations, participants spent a lot of time using the three-dimensional print evidence to decipher what happened and ultimately whether the defendant was guilty: *‘Was it the left? What side was it, the left? Shall I just pick it up?’* (Charley, Jury 2, Line 29). The participants used the evidence to re-enact the crime, using their hands to illustrate guns and physically track bullet trajectories into the skull. Thus, the 3D-printed model appeared to help facilitate understanding as they pieced together what had happened:

I think that the angle of the gun, that it would be unlikely that he would have been able to maintain the position to hold the gun. And, it also said that if he did it himself that it would be right to his head, the gun would be right on his head. Whereas, because there is not as much damage as there would be if that was the case, that means that it was shot from a distance. (Georgia, Jury 1, Lines 13–17)

For these mock-jurors, it seemed that the three-dimensional print was a useful illustrative tool as participants considered whether their own re-enactments correlated with the expert witness testimony. This allowed them to develop their own understanding, rationale, and justification for their decision making. This is seen when Holly (Jury 2, Line 27–28) states, *‘With a suicide, who would clean it [the gun] because he is dead. But where did it come out of though? He was shot in here [moving the skull to point to the entry wound]’.*

Although the mock-jurors expressed real engagement with the three-dimensional prints, their full value as an illustrative tool was perhaps impeded by two participants’ misunderstanding of how the 3D-printed model was generated. The 3D models were created using CT scan data of the victim post mortem, followed by 3D-printing from these data files. However, some participants were seemingly unaware of this: *‘I would get it if the 3D skull was from the actual skull, I mean the body . . .* *it seems that they have gotten the 3D images from pictures, so is it really the same?’* (Georgia, Jury 1, Lines 202–204). This misunderstanding in how the 3D-printed model was developed was shared by a fellow Jury 1 participant, who further suggested that their doubts over the accuracy of the 3D-printed model affected their verdict decisions:

But we have gone with not guilty because although all of the evidence does lean towards guilty, because the way the gun was placed* . . .* , but we do have that doubt that all the evidence was done by pictures. (Jane, Jury 1, Line 214–217)

This scepticism from two of the mock-jurors on the authenticity of the 3D-printed model introduced reasonable doubt and subsequently had a tangible impact on their decision making. This highlights the crucial role that perceived credibility and reliability of the 3D-printed models plays in (mock) jurors’ decisions and underscores the importance of clearly communicating to jurors the rigour associated with the generation of 3D-printed models. As a couple of participants questioned the reliability of the three-dimensional print as evidence, it is important to highlight that the court transcript they were provided with included the expert testimony that the 3D-printed model was a ‘very accurate’. Nonetheless, it could be a limitation of the study that the process of generating the 3D-printed model for the participants was not included in sufficient detail as the transcript only included information that the 3D-printed model resulted from CT scanning of the victim’s skull by a suitably qualified expert. It is therefore essential that pathologists and forensic experts utilise clear, simple and accessible language when presenting a 3D-printed model in court, explaining the technical specifications and industry standards to which they were created. This may prevent jurors from unnecessarily doubting the validity and reliability of the 3D-printed model evidence, as may have occurred here.

#### Theme 4: A piece of the puzzle

As outlined in the previous theme, most participants accepted the 3D-printed model as a valuable illustrative tool, believing the 3D-printed model to provide clarification: *‘So based on the evidence that has been presented . . .* *it is clear that it is not suicide*’ (Tegan, Jury 4, Line 167–168). However, it is noteworthy that all the mock-jurors emphasised that 3D-printed models should only be admitted into court in conjunction with other evidence, as a piece of the puzzle, rather than in isolation:

I think that the evidence is still good evidence* . . .* it is good to see it in real life but is the fact that the other evidence they have presented, also is not from the person or the crime scene.* . . .* I think that you cannot conclude that she is guilty from it. (Georgia, Jury 1, Line 136–138)

Participants highlighted the need for additional evidence to fully understand what had happened to the victim. Some saw the lack of fingerprint evidence as important to the case;

‘And isn’t it weird how Mr. Jones [forensic expert] said that there were no fingerprints on the gun, so if he had committed suicide and he did not have gloves on, he is obviously dead, so how would he be able to clean the gun’ (Alison, Jury 3, Line 218–220)

Other participants focused on the importance of the defendant’s alibi in their decision making, such as Hannah (Jury 3, Line 52) who said: *‘I do not believe that she [defendant] was asleep’*. A reason for this alibi mistrust was provided by Alison (Jury 3, Line 55), who stated: *‘I could not go to sleep after a full-blown argument after being accused of cheating’.* Yet, other mock-jurors trusted the defendant’s alibi account and placed doubt on the accuracy of the coroner’s estimate of time of death, such as Charley (Jury 2, Line 82–83): *‘But it says here the coroner placed the time of death at 3:45, but I believe that he died earlier . . .* *’* and Charley (Jury 2, Line 88): *‘Yeah, she is saying that she had seen him when she came home at 5:15′.*

While the 3D-printed model was considered useful and informative, the mock-jurors also placed significant emphasis on corroborating information, thus suggesting that 3D-printed models used in conjunction with other evidentiary materials hold substantial value for jurors.

### Verdicts

All mock-juries returned a unanimous verdict at the end of their deliberations. This was not the same verdict across all groups as two juries decided on a guilty verdict, and two on a not guilty verdict. However, when looking at the individually expressed beliefs surrounding the defendant’s culpability all jurors except three (Karen, Tegan and Stacey, Jury 4) asserted that they thought the defendant to be guilty but that there was not sufficient evidence to be able to formally return that verdict. For example, Georgia (Jury 1, Line 213) states *‘we went against our actually natural instincts, which is guilty’*. Similarly, Elsie (Jury 4, line 122–124) states:

‘I do not feel like we have enough information to convict her alone, although I do think that she definitely had something to do with it. On the grounds of probability, she probably did do it’.

Thus, group and individual verdicts did not consistently correspond, as can be seen in Table 3.

**Table 3.** Belief in guilt expressed individually within the deliberations and final group verdicts returned.

Jury	Group verdict	Individual belief	Juror
1	Not guilty	Guilty	Katie
		Guilty	Georgia
		Guilty	Jane
2	Guilty	Guilty	Holly
		Guilty	Charley
3	Guilty	Guilty	Hannah
		Guilty	Jessica
		Guilty	Alison
		Guilty	Isobel
4	Not guilty	Not guilty	Tegan
		Not guilty	Karen
		Not guilty	Stacey
		Guilty	Elsie

## Discussion

This study is the first of its kind to utilise a 3D-printed model of an actual murder victim’s remains to provide an in-depth qualitative analysis of mock-jurors’ perceptions and emotional reactions of this evidence. Past research has instead asked the public (as proxy for jurors) their views of hypothetical 3D-printed models, or of 3D-printed models of archaeological rather than forensic remains (for example Carew et al., 2023). The four themes revealed in the analysis – (a) Realistic and creepy, (b) Overcoming uncomfortableness, (c) An illustrative tool, (d) A piece of the puzzle – provide much needed insight into the impact of 3D-printed model evidence in courtrooms, with potential upset, juror misunderstandings of the evidence and ability to re-enact the offence highlighted as key research findings.

### Emotional response to three-dimensional printed reconstructions

Although 3D-printed models are ‘minimally graphic’ compared to gruesome photographs (Edwards & Rogers, 2018, p. 690), these models can be highly detailed due to the technological advances in 3D-printed model practices (Carew & Errickson, 2019). The participant’s perceptions that the evidence was unsettling, as illustrated in the ‘realistic and creepy’ theme, seems to stem from the lifelike and convincing nature of the 3D-printed model. The knowledge held by most participants that the 3D-print was an accurate representation of the victim’s skull, made the evidence unsettling to the participants, rather than the model in and of itself. Thus, it is knowledge of what the model represents – a human life that was violently ended – and the confrontation with these signs of violence that was upsetting to the mock-jurors. Thus, the argument that 3D-printed models in court are equivalent to museum skeletons (Henningsen et al., 2023) does not seem to be supported.

Given the mock-jurors’ perceptions of the realism of the bone-coloured 3D-printed model used in this study, and the process of jury duty being distressing for many jurors (Robertson et al., 2009), suggestions (Villa, 2017) to further increase realism through the introduction of skin colour to models would appear to risk unnecessary additional upset. With up to 50% of jurors worldwide experiencing some signs of trauma following jury duty (Lonergan et al. 2016), potentially increasing the likelihood or severity of these trauma responses seems unwarranted. Dehumanising language has been shown to reduce empathy and moral concern towards refugees (Esses et al., 2003), depression (Reali et al., 2016) and autism (McGuire, 2016). The linguistic choice to use dehumanising language when referring to the 3D-printed model corresponds with the idea of refraining from associating the victim with the evidence, a psychological and linguistic distancing mechanism, which relieves participants of emotional discomfort (Reali et al., 2016). These findings suggest that using colour to enhance the realism of 3D-printed models could make 3D-printed models more disturbing for jurors, without any corresponding benefit in terms of increased evidence understanding (Errickson et al., 2014; Haragi et al., 2020). However, to address this point, future research should quantitively examine the short-term and long-term impact of exposure to these types of 3D-printed models on jurors’ trauma responses.

Past research has posited that empathy and disgust influence jury decision making, as viewing graphic and gruesome photographs resulted in more guilty verdicts in previous research (Salerno, 2017). This falls in line with the culpable model of juror decision making; the realism of three-dimensional prints may enhance victim empathy and feelings of disgust, which in turn encourage higher guilty verdict rates (Molho et al., 2017; Salerno, 2017; Salerno & Peter-Hagene, 2013). Theoretically, 3D-printed models could lead some jurors to experience enhanced victim empathy due to tactile engagement and model realism, suggesting that guilty verdicts may be more common in the presence of potentially emotive 3D-printed models (Skorinko et al., 2014). However, this was not seen in the present study, as empathy for the victim was not overtly expressed within the deliberations, and despite the widespread belief in the defendant’s guilt, half of the mock-juries did not return a guilty verdict. Thus, the results do not appear to support the capable control model (Alicke, 2000). This may be due to the other evidence available in the case and the use of the 3D-printed model as a single piece in a larger puzzle, or doubts over the print’s accuracy reducing the human connection to the victim. Future research that examines empathy and emotional reactions and decision making following exposure to 3D-printed models and alternative evidence presentation mediums – such as photographs and 3D computer models – would address this point.

### Mock-jurors’ interpretations of 3D-printed models

Supporting previous research (Blau et al., 2019; Errickson et al., 2020), the present findings indicate that even if the mock-jurors felt uncomfortable with the 3D-printed model, they still held it in high evidential regard. This is consistent with previous research pertaining to the ‘CSI effect’, whereby jurors expect, trust and rely heavily on scientific evidence in trials due to the influence of crime television shows that depict physical evidence as being inherently reliable (Khanna & Resnik, 2021). Participants used the evidence to reconstruct how the crime may have been carried out, rotating the 3D-printed model and holding ‘finger guns’ to reconstruct the gunshot wound. Participants engaged with the 3D-printed model through multiple modalities (visual, haptic, spatial). As the first qualitative examination of how jurors engage with 3D-printed models, the study lends support to the concept that 3D-printed models aid jurors’ interpretations of courtroom testimonies due to the additional sensory information they provide (Carew et al., 2021). Thus, the participants were able to make sense of the case and re-enact different scenarios to conclude the most plausible way in which the victim had died (Barrera et al., 2019). Subsequently, the 3D-printed model better informed the mock-jurors in their comprehension of evidence, facilitating their decision-making process. The study therefore provides some tentative support for the multimodal learning theory (Bouchey et al., 2021; Massaro, 2012), although the effect of touch versus mere observation of 3D-printed models should be assessed in future research.

Despite their praise for the 3D-printed model’s realism, the participants all expressed that the 3D-printed model alone was insufficient to reach a guilty decision, suggesting the participants were not blinded by the forensic evidence as the ‘CSI effect’ suggests. In fact, previous claims that jurors require multiple forms of implicatory evidence to meet the evidentiary threshold for conviction (Hawkins & Scherr, 2017), was borne out in this study. Although all but three participants (Tegan, Karen and Stacey, Jury 4) in the present study believed the defendant to be guilty (see Table 3), they reported an unwillingness to return a guilty verdict due to the lack of other evidence against the defendant, such as fingerprint evidence at the crime scene. This response was evident despite the presentation of the 3D-printed model in the context of a mock-trial, which provided participants with the wider case evidence. This supports the idea that the quantity of prosecution evidence is integral to reaching the evidentiary threshold (Mancini, 2011; Schweitzer & Nuñez, 2018) and that 3D-printed models do not unfairly sway juror deliberations.

Surprisingly, in contrast to the CSI-effect (Alldredge, 2015), some participants expressed confusion and scepticism over the rigour of the 3D-printed model’s construction, which led to them doubting the accuracy and reliability of the 3D-printed model. The creation of the 3D-printed model for this study used best practice techniques (Carew et al., 2019): scanning the victim’s skull, creating a 3D-printed model, building a model and appreciating that 3D-printed models created in this way are accepted courtroom evidence. Just as a lack of comprehension of forensic evidence led to diminished assessments of the credibility of the expert witness in prior research (Wilcox & NicDaeid, 2018), the current study reveals that uncertainties regarding the reliability of the 3D-printed model influenced evaluations of its credibility and usefulness. This shows the need for good quality verbal evidence provided in accessible lay-terminology to accompany 3D-printed models in court. The verbal evidence must not only outline the features of the physical trauma on the 3D-printed model, but also clearly outline the rigorous standards and qualifications associated with the creation and presentation of each 3D-model provided as evidence (Wilcox & NicDaeid, 2018). Certainly, implementation of these specifications is contingent on universal acceptance and implementation of standards for use of 3D-printed models in forensic settings (Carew et al., 2019). The findings further suggest that introducing a mechanism to check whether jurors have correctly understood this technical evidence would be highly worthwhile, such as a quality evidence summary to help jurors make sense of technical information (Wilcox & NicDaeid, 2018).

The reactions captured within this project highlight how 3D-printed models can serve as a focal point in evidence presentation and allow mock-jurors to engage with the evidence more deeply, putting complex forensic language into a visual perspective. The presence of the 3D-printed model did not prevent participants from discussing other case evidence in detail, although they were able to be critical of the accuracy of the 3D-printed model, seeing it as a useful tool as part of a wider puzzle of evidence.

### Limitations

The sample in the present study was small and consisted of undergraduate students. Although the small sample size could limit the generalisability of the findings, mere sample size is not an indicator of quality in qualitative research, with data richness the primary factor to consider (Braun & Clarke, 2022; Malterud et al., 2016; Terry et al., 2017). The exploratory nature of the research and the new themes found within the data provide valuable and tangible avenues for future researchers and practitioners to examine (Hammersley, 2015; Park et al., 2017). Information power (Materud et al., 2016) is relevant here: the aims, sample specificity, analysis strategy and established theory all would suggest a larger sample; however, the quality of dialogue supports a smaller sample. Similarly, although data saturation is not a goal of reflexive thematic analysis (Braun & Clarke, 2021), in line with common thematic analysis practice, analysis of the transcripts was halted once analysis of the data failed to result in new themes being discovered, and ‘saturation’ was achieved. Inviting participants to check the transcript post deliberation could help confirm transcript accuracy (although two authors were involved in the transcription to reduce inaccuracy) and confirm the appropriateness of the themes and interpretation provided. That the study offers new information that contributes to current understanding via saturation suggests that appropriate sample size was employed.

Moreover, student samples remain common in the mock-juror academic literature (Krauss & Lieberman, 2017; Wiener et al., 2011) as student participant decision making in mock-juror studies has been shown to correspond with that of the wider public (Bornstein et al., 2017). However, the small sample undoubtably limits the generalisability of the findings, and in line with the stepped research recommendations of Curley and Peddie (2024), future research should build on the initial student sample used here and explore the perceptions of mock-jurors within a larger and more varied community sample.

Although both males and females were eligible to participate, only females volunteered to participate within the project, and it has been demonstrated that females find jury duty more stressful than males (Robertson et al., 2009). The more prolific stress response amongst female jurors is likely to have multifaceted origins – for example, juror stress is more likely where the trial content is congruent with any trauma the jurors themselves had experienced in the past (Brooks et al., 2025; Robertson et al., 2009), and the role of this is unknown. Exploring the role of prior traumatic experiences of both males and females in their evaluation of 3D-printed model evidence would therefore be a valuable addition to future research in this area. Notwithstanding this fact, the study did not seek to examine whether gender differences exist in how evidence is evaluated. Instead, we aimed to examine the emotional reactions and experiences of mock-jurors in response to 3D-printed models. Having a sample of those (mock) jurors that are more likely to experience distress following jury service in general (females; Robertson et al., 2009) whilst limiting generalisability, provides the unanticipated benefit of examining the experience of this type of evidence amongst those who are most vulnerable to distress without their experiences being overshadowed by those who may be less likely to find this evidence (or indeed any evidence) distressing. This certainly means that we cannot generalise these findings to all jurors (though that was never the intention of the paper), rather we argue that the paper provides us with a new perspective and in-depth understanding of how female mock-jurors perceive 3D-printed models of human remains. Nevertheless, future research would benefit from inclusion of both male and female participants.

The present research utilised a 3D-printed replica of a human skull as this was the evidence available to the research team following a request for a 3D-model of a murder victim’s remains. The skull is arguably the component of the human skeleton that is most easily recognisable as human to non-experts. It is plausible therefore that juror emotional reactions to the 3D-printed model skull may be more pronounced than reactions to a 3D-printed model femur, for example, which could more easily be imagined as belonging to another species (Gollapalli et al., 2021). Similarly, the ‘humanness’ of the skull may more readily facilitate visualisation of the individual victim in the case, leading to this evidence being perceived as particularly gruesome and graphic (Haragi et al., 2020) and raising empathy towards the victim (Lilley et al., 2023), compared to a 3D-printed model of a different skeletal component (such as the pelvis or radius, for example). Moreover, as the first study of its kind, it is unclear whether other features of 3D-printed models such as characteristics of the victim (child vs. adult), physical trauma type (gunshot, knife, blunt force trauma, etc.), or the number of skeletal components (one model vs. several different 3D-printed models showing physical trauma to numerous skeletal components) may affect (mock) juror perceptions and emotional reactions. Thus, future research should build on these findings to explore mock-juror responses to a range of 3D-printed models of human skeletal remains to help establish best practice in presenting evidence of this type in court.

A limitation of the research was the lack of information provided to the participants about the process of generating the 3D-printed model. As mentioned previously, some participants appeared to misunderstand how the evidence was generated. Nonetheless, the findings suggest that future research should provide detailed technical information on 3D-model generation to replicate real-world practice and reassure jurors that the 3D-printed model is accurate and reliable, and that similarly generated models have been considered reliable enough to be utilised as demonstrative evidence in other criminal trials in England. Future research should assess mock-jurors’ comprehension and acceptance of this technical information to determine appropriate levels of detail needed in these circumstances.

A final overarching limitation to the research was the reliance on a mock-trial paradigm. Participation in the study was brief and not comparable to the days, weeks or even months duration of real-world murder trials. Similarly, a written transcript of court proceedings is vastly different to the experience of seeing and hearing evidence live in court and the associated understanding that the verdict returned will profoundly affect the lives of many. Despite these limitations, mock-jury research is commonplace in the psychological literature (for example, Richardson et al., 2025; Wiener et al., 2011) as practical considerations and legal restrictions in England and Wales prevent deliberations from actual trials being utilised in academic research (Contempt of Court Act 1981 section 20D). Despite the limitations associated with jury replication research, they do provide a useful indication of how potential jurors (all participants were jury eligible) perceive and utilise this novel evidence. Moreover, the present research compares favourably against three of the four markers (sample representativeness, material realism, inclusion of deliberation and seriousness of participant engagement) of generalisable mock-jury research (Ormston et al., 2019). Future research should aim to explore the themes generated in this study by means of an interview or questionnaire of former jurors.

### Summary and implications

This novel study investigated mock-jurors’ interpretations and emotional reactions to 3D-printed models of human skeletal remains. In terms of interpretations (Research Question 1), these 3D-printed models are a useful tool and piece of the puzzle for (mock) jurors to understand injuries, re-enact scenarios and, in conjunction with other case evidence, reach verdicts. Nonetheless, when considering mock-jurors’ emotional reactions to this type of evidence (Research Question 2), uncomfortableness and fear were evident in the mock deliberations. As 3D-printed models are routinely being used in courts around the world, we urge caution and careful case-by-case consideration as to whether these additional visual aids are necessary in promoting juror understanding over and above pathologists’ testimony. Further research and collaboration between legal professionals, psychologists, pathologists and forensic professionals is required to ensure that legal guidance pertaining to the creation and use of 3D-printed models recognises not only the necessary technical standards, but also the ethics and practicality of their introduction to jurors in court. Development of these professional standards for utilising 3D-printed models in courtroom settings should be situated within the context of a trauma-informed courtroom where the emotional risk to jurors (and others present), not solely the practical utility of 3D-printed models, should be a primary consideration in the presentation of 3D-printed models of human skeletal remains in court.
